# Global neurosurgical workforce density—you cannot improve what you do not measure

**DOI:** 10.1186/s41016-021-00252-2

**Published:** 2021-08-01

**Authors:** Ulrick Sidney Kanmounye, Adam Ammar, Myron Rolle, Abdessamad El Ouahabi, Kee B. Park

**Affiliations:** 1 Global Neurosurgery Initiative, Program in Global Surgery and Social Change, Harvard Medical School, 641 Huntington Avenue, Boston, MA 02115 USA; 2 Department of Neurosurgery, Montefiore Medical Center, University Hospital for Albert Einstein College of Medicine, New York, NY USA; 3 Department of Neurosurgery, Massachusetts General Hospital, Boston, MA USA; 4 Department of Neurosurgery, CHU Mohammed V, Rabat, Morocco

**Keywords:** Global neurosurgery, Global surgery, Low- and middle-income countries, Neurosurgery, Workforce density

## Abstract

Five million neurosurgical cases go untreated each year. This is in part due to the lack of neurosurgical care providers. The World Federation of Neurosurgical Societies has spearheaded efforts to monitor the number of neurosurgical providers around the globe since 2016. In this perspective, we discuss why, when, and how the neurosurgical workforce should be measured.

## Introduction

In 2015, the Lancet Commission on Global Surgery published a report on disparities in the burden of surgical diseases and surgical system resources [4]. In this report, the commissioners proposed six global surgery targets to measure a surgical system’s capacity to provide safe, affordable, and timely surgical care. One of the six targets measured the surgical, obstetric, and anesthetic specialist workforce density. Within the global surgery milieu, neurosurgeons organized and defined priorities for the global neurosurgery movement [5, 6]. One of those priorities was to increase the neurosurgical workforce density.

## What is neurosurgical workforce density?

Neurosurgical workforce density is defined as the number of neurosurgeons per population. To monitor the evolution of the neurosurgical workforce, the Global Neurosurgery Committee of the World Federation of Neurosurgical Societies proposed the tracking of workforce densities across the globe as one of its goals [6]. According to Dewan et al. [4], at least 5 million neurosurgical cases are left untreated, mostly due to a neurosurgical workforce deficit, with an estimated 23,218 additional neurosurgeons needed to address this case deficit [4]. Further studies had a focus on neurotrauma. The rationale for defining neurotrauma was simple: neurotrauma constitutes 52% of the global burden of neurosurgical diseases, and 74% of the burden of neurotrauma is borne by low- and middle-income countries [4]. In a comprehensive policy document for the management of traumatic brain and spine injuries, Corley et al. defined the target neurosurgical workforce density and calculated the minimum workforce density necessary for adequate management of neurotrauma in the world [2]. The threshold was found to be 1 neurosurgeon per 212,000 people.

## Why should we measure neurosurgical workforce density?

The neurosurgical workforce density depends on two variables: the absolute number of neurosurgeons and the population in a given geographical area. The absolute number of neurosurgeons depends on neurosurgeon training, retirement, emigration, and mortality rates. Similarly, the population depends on natality, immigration, emigration, and mortality rates. As a result, a simple headcount of neurosurgeons does not accurately describe access to neurosurgical providers. This is especially important in geographical regions experiencing migration, pandemics, or natural disasters. The neurosurgical workforce density gives a better estimate for a region, and the more the data is disaggregated, the more reliable it is.

Disaggregated data can help identify populations that are disproportionately affected by neuropediatric, neurovascular, neuro-oncologic, spine, and functional diseases. These diseases cause significant morbidity and mortality especially when access to neurosurgical care is limited [3]. Timely access to neurosurgical care is essential for the management of neurotrauma. In low- and middle-income countries, the golden hour for traumatic brain injury is 4 h [1]. Hence, neurosurgical workforce density can inform the policymakers and complement nationwide strategic plans to expand the number of neurosurgical centers so no patient lives more than 4 h away from the point of definitive care. These neurosurgical centers should be staffed by neurosurgical care providers (specialist or non-specialist).

In low- and middle-income countries, as a result of the enormous workforce deficit and lack of resources in rural areas, care is often provided by non-specialist neurosurgical providers [7]. Non-specialist surgical providers often practice life-saving interventions but should consult specialist neurosurgical providers to decide on the appropriate management of cases [8].

## Where should we measure neurosurgical workforce density?

For the reasons mentioned above, the neurosurgical workforce density should be tracked, at a minimum, at national levels in all countries. This is the only way to identify countries with severe neurosurgical workforce deficits. And, as soon as feasible, the distribution of the neurosurgeons within a given country should be measured by tracking the regional and district level neurosurgical workforce densities. Granular metrics at the local level is vital to exposing and addressing disparities in access to neurosurgical care that exist within the borders of any given country, typically along socioeconomic lines.

## Who should measure neurosurgical workforce density?

Global professional organizations like the World Federation of Neurosurgical Societies (WFNS) have the authority, duty, and resources to undertake, coordinate, and disseminate these metrics. Fortunately, the leadership of the WFNS shares this point of view and, through its Foundation, has supported data collection and mapping of neurosurgical workforce density by the Global Neurosurgery Initiative (Program in Global Surgery and Social Change, Harvard Medical School) since 2016 [9]. This collaboration has led to the wide dissemination of the results of the project that have informed global neurosurgery policies and initiatives. The success of this project is a testament to the role and potential of partnerships between professional organizations and academic institutions in achieving equity in neurosurgical access.

## How should we report neurosurgical workforce density?

Neurosurgical workforce density data collection is time-intensive and difficult to maintain by a single academic institution in the long run. Ideally, a mechanism is needed for self-reporting by each country into a common database kept at the WFNS Headquarters. By shifting the responsibility of measuring the neurosurgical workforce density to each country, and thereby having each country take ownership, the accuracy would be improved and sustainability ensured. The WFNS could encourage communication and knowledge-sharing between professional societies at the local, regional, and global levels through the provision of a unified and harmonized data collection and reporting platform and leveraging of social media outlets. Such coordination of data collection efforts will limit redundancy and minimize survey fatigue among respondent neurosurgeons.

## Conclusion

The collection, analysis, and tracking of neurosurgical workforce densities globally are critical to the attainment of universal neurosurgical care. The global neurosurgical community, through the WFNS, needs to invest in the logistics of data collection to streamline the process. If this is done, we can increase respondent rates, accuracy, and considerably cut down delays.

When you can measure what you are speaking about, and express it in numbers, you know something about it.

- William Thomson

If it can be measured, it can be changed

- Doug Pratt

## Data Availability

Not applicable.

## Abbreviations

WFNS: World Federation of Neurosurgical Societies
